# Supplementary material for: Insight into RNA–DNA primer length counting by human primosome
Source: Nucleic Acids Res. 2022 Jun 11;50(11):6264–70. doi: 10.1093/nar/gkac492 (PMC9226528; doi:10.1093/nar/gkac492)
Supplement: gkac492_Supplemental_File [file gkac492_supplemental_file.pdf]

SUPPLEMENTARY DATA
for the article

Insight into RNA-DNA primer length counting by human primosome

Andrey G. Baranovskiy[1], Alisa E. Lisova[1], Lucia M. Morstadt[1], Nigar D. Babayeva[1], Tahir H. Tahirov[1]

[1]Eppley Institute for Research in Cancer and Allied Diseases, Fred & Pamela Buffett Cancer Center. University of Nebraska Medical Center, Omaha, NE, USA.

Corresponding author Tahir H. Tahirov (ttahirov@unmc.edu).

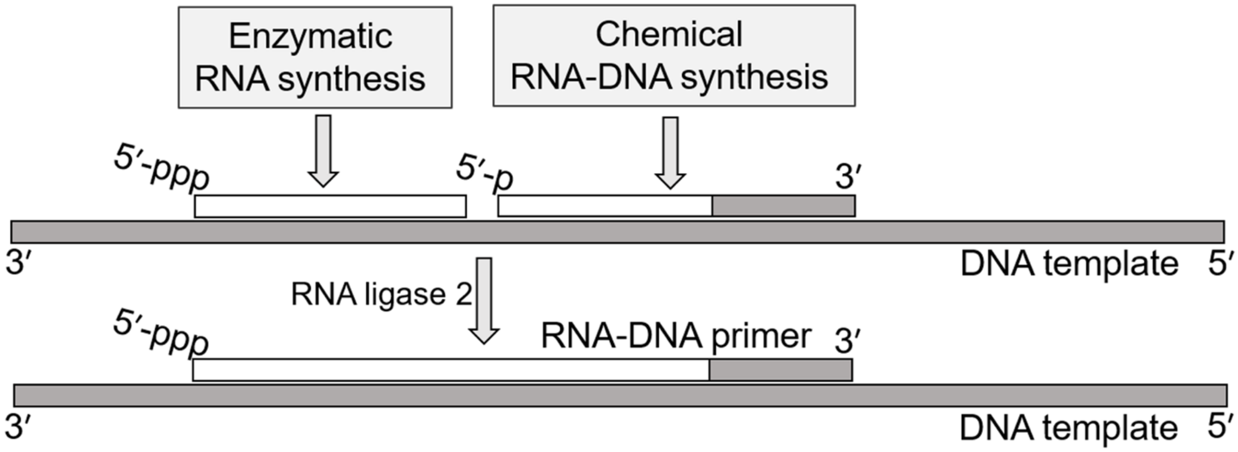

Supplemental Figure 1. A scheme of 12-mer RNA-DNA primer synthesis. The 5-mer RNA primer with a 5′-triphosphate was generated by RNA polymerase of bacteriophage T7. The 7-mer RNA-DNA with a 5′-phosphate and three dNMPs at the 3′-end was synthesized by IDT Inc. Both primers were annealed to a DNA template and ligated by RNA ligase 2 of bacteriophage T4 (New England BioLabs Inc.). White and gray blocks represent RNA and DNA, respectively.

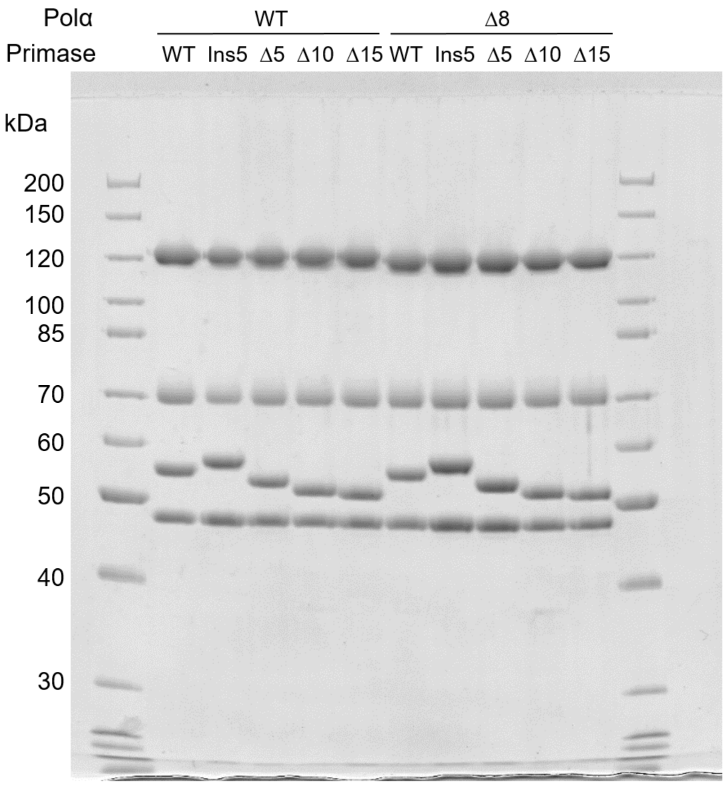

Supplemental Figure 2. The purity of primosome and its mutants. Proteins were separated by 8% SDS-PAGE and stained by Coomassie Brilliant Blue R-250. Modifications in linkers of p58 (L1) and p180 (L2) are shown above the wells. A disordered N-terminus of p180 (residues 1-334) is deleted.

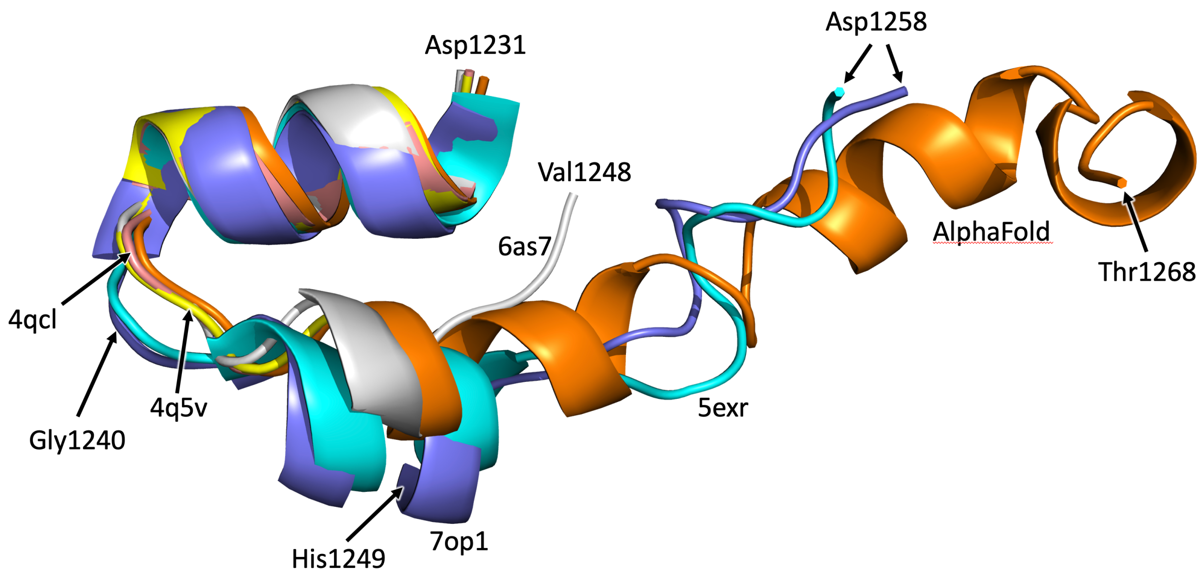

Supplemental Figure 3. Comparison of Polα structures revealing different conformations or partial disorder of p180 residues 1240-1249. These residues precede the linker L2 (1250-1267). PDB accession numbers of used coordinates are indicated. The L2 structure predicted by AlphaFold is also used for alignment.

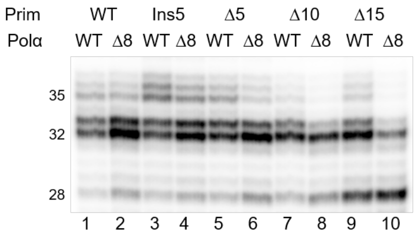

Product length	Integrated density									
	lane 1	lane 2	lane 3	lane 4	lane 5	lane 6	lane 7	lane 8	lane 9	lane 10
35-37 (A)	6217	5262	10678	9815	7864	4701	2695	1546	4388	1276
32-33 (B)	13862	19207	13810	21743	18423	20893	13843	13032	17673	8644
28 (C)	2010	3211	2267	3461	2995	4609	2693	4845	8451	10759
ratio A/B	0.45	0.27	0.77	0.45	0.43	0.23	0.19	0.12	0.25	0.15
ratio C/B	0.15	0.17	0.16	0.16	0.16	0.22	0.19	0.37	0.48	1.24

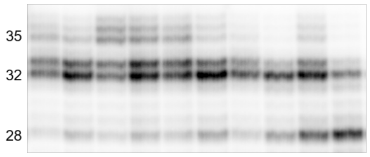

Product length	Integrated density									
	lane 1	lane 2	lane 3	lane 4	lane 5	lane 6	lane 7	lane 8	lane 9	lane 10
35-37 (A)	5917	6474	12770	9780	7752	4755	2867	1439	4433	1223
32-33 (B)	14989	24741	17816	22921	19561	21889	17729	14175	19345	8922
28 (C)	2112	4366	3012	3942	3342	5226	3557	5436	9725	11680
ratio A/B	0.39	0.26	0.72	0.43	0.40	0.22	0.16	0.10	0.23	0.14
ratio C/B	0.14	0.18	0.17	0.17	0.17	0.24	0.20	0.38	0.50	1.31

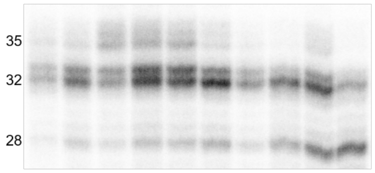

Product length	Integrated density									
	lane 1	lane 2	lane 3	lane 4	lane 5	lane 6	lane 7	lane 8	lane 9	lane 10
35-37 (A)	2296	3098	6915	7843	6976	3723	1548	1262	3311	932
32-33 (B)	6299	11375	9246	17632	16335	15743	8388	10801	13791	6855
28 (C)	914	2032	1574	3046	2886	3717	1763	4056	6575	7697
ratio A/B	0.36	0.27	0.75	0.44	0.43	0.24	0.18	0.12	0.24	0.14
ratio C/B	0.15	0.18	0.17	0.17	0.18	0.24	0.21	0.38	0.48	1.12

Supplemental Figure 4. Quantification of gels corresponding to Figure 3. The integrated densities of selected bands were quantified using ImageJ software.
